# Commentary: Examining contextual factors contributing to differentials in COVID-19 mortality in U.S. vs. India

**DOI:** 10.3389/fpubh.2022.995751

**Published:** 2022-11-01

**Authors:** Preeti Pushpalata Zanwar, Katrine L. Wallace, Christopher Soria, Arokiasamy Perianayagam

**Affiliations:** ^1^Jefferson College of Population Health, Thomas Jefferson University, Philadelphia, PA, United States; ^2^National Institutes on Aging (NIA) Funded Network on Life Course Health Dynamics and Disparities, University of Southern California, Los Angeles, CA, United States; ^3^School of Public Health, University of Illinois Chicago, Chicago, IL, United States; ^4^Demography Department, University of California, Berkeley, Berkeley, CA, United States; ^5^International Institute for Population Sciences, Mumbai, India; ^6^National Council of Applied Economic Research (NCAER), New Delhi, India

**Keywords:** aging, COVID-19 mortality, excess mortality, disparities, inequalities, COVID-19 pandemic, population demographics

## Introduction

As of July 2022, the cumulative, confirmed COVID-19 mortality rates in the United States (U.S.) and India are roughly 3,000 and 370 deaths per 100,000 population, respectively (1). Rates are far lower in India vs. the United States, despite the U.S., dedicating 14% more of their gross domestic product (GDP) on healthcare spending than India (Table 1). However, COVID-19 mortality rates in India have been highly contested. COVID-19 mortality in India has been referred to as the “Indian death paradox” (2) because the death rate is disproportionately low relative to the number of COVID-19 cases the country has experienced. We examine the two most evidence-based and plausible explanations of India's low death rates during the COVID-19 pandemic, which are (1) demographic dynamics and (2) factors contributing to increasing divergence in COVID-19 mortality in the U.S. vs. India, including the systematic undercounting of deaths in India.

**Table 1:** Economic and demographic inequalities in the United States vs. India.

**Population** **characteristics**	**United States**	**India**
*Economic inequalities*		
Income classification	High-income	Lower middle income
GNI per capita, USD (2021)	70,430	2,170
GDP spent on healthcare (%)	17	3
Overall development (United Nations)	Developed	Developing
*Demographic inequalities*		
2022 Population	334.81 M	1.41 B
Men (n, %)	162.76 M, 48.6	730 M, 52
Women (n, %)	171 M, 51.1	675 M, 48.1
Age > 65 years (n, %)	56 M, 16.9	98 M, 7.0
Life expectancy in years		
Overall	79.1	70.4
Female: Male	81.7: 76.6	71.8: 69.2
Age dependency ratio (2021)^*^	55	48
Fertility rate (births per women) (2022)	1.782	2.159

Notes: M, Millions; B, Billions, GNI, Gross National Income; GDP, Gross Domestic Product.

* Age dependency ratio (% of working-age population).

Source: https://worldpopulationreview.com/country-rankings/high-income-countries.

https://www.worldometers.info/demographics/life-expectancy/.

https://data.worldbank.org/indicator/SP.POP.DPND?locations=IN.

https://www.statista.com/statistics/737923/us-population-by-gender/.

https://www.theglobalstatistics.com/india-population-statistics/#:~:text=In%202022%2C%20males%20make%20up,total%20population%20at%20675%20million.

https://www.americashealthrankings.org/explore/senior/measure/pct_65plus/state/ALL?edition-year=2022.

## Demographic dynamics

Cumulative global COVID-19 data show older adults ages 65 years and above, and older men, to be extremely vulnerable to COVID-19 fatality. Early in the pandemic, 8 out of 10 COVID-19 reported deaths in the United States were in older adults 65 years and above (3). Overall, the age gradients of COVID-19 mortality are steeper in the low mortality settings of high-income countries. The case fatality rate (CFR) of COVID-19 for older adults ages 65 years and above is 15 times higher than for younger adults (3, 4). Low-and middle-income countries display relatively moderate age gradients of COVID-19, where population age structures are comparatively young. In India, data released by the Ministry of Health and Family Welfare (MoHFW) show the age-wise share of COVID-19 deaths at older ages (i.e., 60 years and above) is ~50%, with 46% of deaths occurring in the prime adult ages of 30–60 years, with a median age at death of 60 years. This is almost 20 years lower than what applies in high-income countries. Although India's death toll is large due to the sheer size of its population, its COVID-19 mortality rate per capita is relatively low due to a relatively young age structure. Currently, only about 7% of India's population is 65 years and older. By 2050, however, that number is expected to approximately double to about 15%, and double again to ~30% by the year 2100 (5). Since the risk of dying of COVID-19 increases with age globally and is greatest in older adults, one plausible theory is that India, with a younger demographic makeup, has a lower risk of death on a population level.

Additionally, other demographic characteristics, such as gender, contributed to cross-national COVID-19 death rate disparities. In the United States, as of September 2022, 55.1% of COVID-19 deaths were in men, 44.9% of COVID-19 deaths were in women while COVID-19 death counts for other sex and gender minorities are not available (6). Similarly, in India, as of 18 May 2021, 64% of deaths were in men while 36% of deaths were in women, with no data on other sex and gender minorities (7). As of 9 April 2022, the male to female ratio for deaths in the U.S. among confirmed cases was 1.39, with the oldest aged men (80 plus years) at the greatest disadvantage for COVID-19 deaths, followed by women of 80 plus years. However, early in the pandemic in India, men were at a greater disadvantage than women with a case fatality rate (CFR) of 3.3 and 2.9%, respectively, where the CFR was estimated as the ratio of confirmed deaths to the total number of confirmed cases (8).

## Causes and factors contributing to differentials in COVID-19 incidence and fatality

Globally, the age pattern of the COVID-19 pandemic cases points to a near normal distribution with three-fifths of the cases concentrated in adults aged 30–65 years. In contrast, the fatality rate is very high for older adults aged 60 years and above compared with younger adults aged < 60 years. Second, the age gradients of COVID-19 mortality demonstrate sharp differences between high- and middle-income countries; with stepper age gradients for high-income countries compared with moderating age gradient for lower middle-income countries, such as India. Third, biological sex differences are shown to contribute to excess male COVID-19 deaths at older ages. However, data suggest gender-associated risk of exposure may affect rates of infection and fatality differently for men and women (9). Fourth, during the pandemic, the age-associated chronic diseases has taken millions of lives annually and a larger share of younger lives ages 30–70 years in low- and middle-income countries (LMIC's) comprise the global burden (10). The premature onset of chronic health conditions among older adults aged 45 years and above is more common in LIMCs with workforces in these ages exposed to heightened risks of contracting COVID-19; this shifts the share of COVID-19 mortality from older adult ages to younger adult ages of 30–60 years. About 90% of COVID-19 deaths of older adults are assigned causes associated with pre-existing chronic health conditions, such as cardiovascular diseases, diabetes, kidney disease, respiratory system disease, and cancer (11, 12). Finally, the huge humanitarian and economic crisis of the COVID-19 pandemic led to a disproportionate burden on the health and mortality impact on the poor and vulnerable, bringing existing socioeconomic disparities in health into sharper focus.

## Socioeconomic disparities in COVID-19 incidence and fatality

Across the world, COVID-19 incidence and fatalities are distributed unequally among those with different levels of material and social deprivation (13). People living in congested poor urban settlements, migrant wage earners in low- and middle-income countries, racial minorities in high-income countries, and the frontline healthcare workforce at the bottom of the hierarchical spectrum have borne a disproportionately heavy burden of infection and death. The COVID-19 pandemic has brought such existing socioeconomic health disparities into sharper focus both in the United States and in India (14). India and the United States have different and unique underlying socioeconomic and structural determinants across many domains and levels of influence (9, 15–19). These so called upstream drivers of disparities rooted in historical, social, political, and cultural determinants have intersected during the COVID-19 pandemic and have amplified and exposed the underlying and existing disparities within specific country contexts, thereby creating inequities in COVID-19 mortality. The demographic and socioeconomic disparities in the health gradient provide an important framework to deepen the understanding of, and to mitigate, the health equity effects of the disease (13). According to the OXFAM International report on extreme inequality numbers in India, the lack of universal health coverage in India is one of the drivers of socio-economic inequalities in the health sector, which disproportionately affects health outcomes of marginalized communities (20).

Additionally, the waves of COVID-19 lockdown and related measures across States and Union Territories in India have led to state variations in COVID-19 incidence and deaths (Table 1). There has been a huge humanitarian and economic crisis with heavier burdens of COVID-19-related health consequences for the poor and vulnerable. Excess mortality was greater in rural, less affluent areas in India. With the worldwide healthcare infrastructure and human resources of health set exclusively for COVID-19 priority during the lockdown, people in urgent/emergency need of critical healthcare for pre-existing chronic health conditions, acute conditions, and maternity and childcare, faced either no access to care or extremely limited access. This led to a sizeable number of deaths from other causes; most countries reported disruption to healthcare services for non-communicable diseases (NCDs) (12).

## Undercounting of deaths in India

Measuring mortality in India is difficult. About half of India's deaths occur at home (21). Of 10 million deaths every year, over 3 million are not registered and over 8 million have not undergone medical certification (22). A quarter of women's deaths are not counted (23) and registration is especially low in the poorest states, such as Uttar Pradesh and Bihar (24).

In contrast, the completeness and accuracy of the US mortality reporting are generally more reliable, even during the COVID-19 pandemic (25). However, even in the United States, COVID-19 deaths have been somewhat undercounted in certain areas of the country, namely, rural areas and in the southern states (26). The Centers for Disease Control and Prevention (CDC) estimates that overall U.S. excess deaths between March 2020 and March 2022 totaled 1,105,736, which was only 15% more than the 958,864 official death toll reported from COVID-19 over that period (3).

Methodologically, excess mortality is the best way to estimate the total net mortality burden of the COVID-19 pandemic. Excess mortality is the difference between the *observed* deaths and the *expected* number of deaths during the same time period, based on data from years before the pandemic. Thus, the “excess deaths” estimate the extra number of deaths from all causes during the pandemic relative to what would have been expected, had the pandemic not occurred.

In May 2022, the World Health Organization (WHO) released a report estimating the global mortality burden of the COVID-19 pandemic to be 14.9 million excess deaths, in contrast to the 5.4 million COVID-19 deaths that had been officially reported. This report claims, of these deaths, a cumulative 4.7 million deaths due to COVID-19 occurred in India alone (27), whereas India's official estimates place the total at ~525,000 as of July 2022 (22). Furthermore, two other large studies of deaths in India during the COVID-19 pandemic also estimated India's excess mortality to be 6–8.3 times higher than expected (22, 24), with much variability in the ratios of observed to expected between Indian states (from 0.96 in Goa to 26.7 in Bihar) (24). For a more realistic count of excess mortality, researchers must wait until all-cause mortality data for the COVID-19 period (March 2020 to December 2021) is released from the Sample Registration System (SRS), possibly in 2024.

## Conclusion

**Figure 1:**
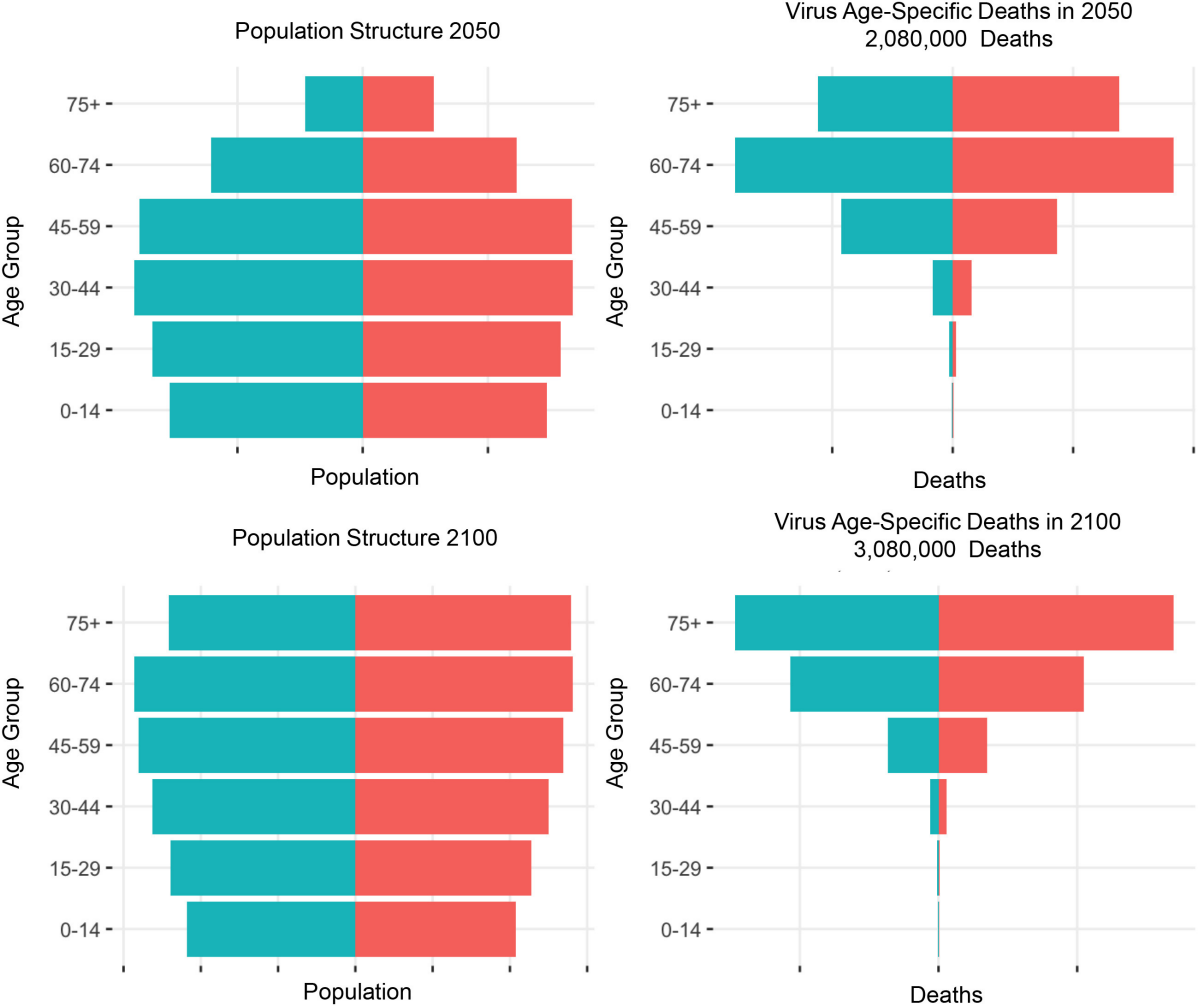
Population projections of age structure and virus age-specific deaths in India. United Nations. Department of Economic and Social Affairs, Population Division, World population prospects 2022, Source: https://population.un.org/wpp/Download/Standard/Population/.

A future pandemic is inevitable. A pandemic caused by a virus with mortality rates similar to severe acute respiratory syndrome coronavirus-2 (SARS-CoV-2), which kills proportionately more older people in a pattern that follows “Gompertz' Law” (28), would become increasingly deadly as India's falling fertility rates, negative net migration of mostly young people, and rapidly growing life expectancy (29) and rising longevity produce proportionally older populations over time (Figure 1). The same “COVID-19-like” virus scaled to kill 1 million people in India in 2022 would kill just over 2 million in 2050, and 3 million in 2100. Thus, changes in age structure alone would result in more deadly pandemics in the near and distant future. A potential limitation of this assessment, and of health information in India in general, is the lack of consistency and completeness in public health data and vital statistics. The contrast between the United States and India's excess mortality highlights these disparities in the public health infrastructure, limited medical care for critically ill patients in a large-scale health crisis, and a lack of vital recordkeeping capacities. Before the next pandemic, developing nations, such as India, need to prioritize investments in more resilient health systems that can sustain essential health services during crises, including stronger health information systems.

## Author contributions

PPZ drafted Table 1. CS contributed to Figure 1. KLW and PPZ revised the commentary. PPZ, KLW, CS, and AP contributed to the conception and writing of this commentary. All authors contributed to the article and approved the submitted version.

## Conflict of interest

The authors declare that the research was conducted in the absence of any commercial or financial relationships that could be construed as a potential conflict of interest.

## Publisher's note

All claims expressed in this article are solely those of the authors and do not necessarily represent those of their affiliated organizations, or those of the publisher, the editors and the reviewers. Any product that may be evaluated in this article, or claim that may be made by its manufacturer, is not guaranteed or endorsed by the publisher.
